# Gallstones top to toe: what the radiologist needs to know

**DOI:** 10.1186/s13244-019-0825-4

**Published:** 2020-02-05

**Authors:** M. C. Murphy, B. Gibney, C. Gillespie, J. Hynes, F. Bolster

**Affiliations:** Department of Radiology, Mater Misericordiae University Hospital, Eccles St, Dublin 7, Ireland

**Keywords:** Gallstones, Gallbladder, Biliary, Calculus, Pathology

## Abstract

Gallstone-related disease can have significant associated morbidity and mortality worldwide. The incidence of gallstone-related disease in the Western world is on the increase. There are multiple different pathological manifestations of gallstone disease: the presentation, diagnosis and associated complications of which vary significantly depending on anatomical location. The role of imaging in gallstone-related disease is broad with radiology playing an essential role in the diagnosis, management and follow-up of gallstone-related pathologies. This paper distills the broad range of gallstone-related pathologies into an anatomical map, discussing the disease processes involved at each point along the biliary tree and reviewing the strengths and weaknesses of different imaging modalities for each distinct disease process.

## Key points

- Gallstone-related pathology is on the increase in the Western world.
- Gallstones can be located within the gallbladder, migrate into the biliary tree or outside the pancreaticobiliary system altogether with associated pathology.
- Imaging of gallstones and associated pathology requires a multimodality approach.

## Introduction

Gallstones are solid rounded particles composed of a combination of cholesterol and bilirubin that form within the gallbladder and within the biliary system. The size and number of gallstones is variable with some patients forming multiple small gallstones and others forming single or few large stones.

The incidence of gallstones is increasing in Western populations as obesity levels rise. In the USA, 8.6% of Caucasian men and 16.6% of women have gallstones [1]. The vast majority of gallstones are asymptomatic and require no follow-up; however, approximately 10–15% of gallstones will become symptomatic over a period of 10–15 years of follow-up [2, 3]. The symptomatic manifestations of gallstones are variable and range from mild symptoms such as biliary colic to severe acute presentations such as pancreatitis, which can be associated with significant morbidity and mortality. In a patient that has suffered a symptomatic manifestation of gallstones, the incidence of a further manifestation over their lifetime is approximately 3% per year [2]. The risk for gallstone-related pathology is also related to the number and size of stones with numerous larger stones more likely to cause symptoms. For this reason, it is suggested that once symptoms present, the patient should be offered a cholecystectomy [3–5].

The main risk factors for gallstone formation are outlined in Table 1, and the factors protective against gallstones are outlined in Table 2.

**Table 1:** Main risk factors for gallstone formation

Risk factors for gallstone formation	Comment
Age	The incidence increases with age but symptomatic presentation is most common in middle age.
Gender	More common in females by a ratio of 2:1.
Race	More common in Western, Caucasian, Hispanic and Native American populations.
Family history	A first degree relative with a history of gallstones doubles the risk.
Obesity	Increased risk of cholesterol stone formation.
Rapid weight loss	Bile stasis due to reduced calorie intake and increased cholesterol mobilisation.
Haemolysis	There is an increased incidence of associated with haemolytic disorders such as sickle cell disease and the thalassemias.
Oral contraceptives and oestrogen replacement therapy	
Pregnancy	
Raised serum lipids	Increased risk of cholesterol stone formation.
Raised serum bilirubin	Increased risk of pigmented stone formation.
Cirrhosis	
Gallbladder stasis	Stasis of flow allows stones time to form.
Diabetes mellitus	Insulin resistance increases circulating cholesterol.
Crohn’s disease	
Certain medications

**Table 2:** Factors protective against gallstones

Factors protective against gallstone formation	Comment
Statins	Reduce bile cholesterol concentration
Ascorbic acid	Alters cholesterol catabolism
Unsaturated fats	Alters bile acid composition
Coffee	Alters cholesterol catabolism
Vegetable proteins and nuts	Increase ascorbic acid levels

There are three main types of gallstones: cholesterol, mixed and pigmented. Cholesterol gallstones account for 10% of gallstones and are composed of at least 50% cholesterol and form with supersaturation of bile. Patients with high-fat diets and high serum lipids are more likely to have a higher cholesterol composition. Pigmented gallstones, which are darker in colour and are composed mainly of bilirubin with < 20% cholesterol, form with supersaturation of unconjugated bilirubin. There are two subtypes of pigmented gallstones: black pigmented gallstones which are formed due to chronic haemolysis, cirrhosis or intestinal malabsorption, and brown pigmented gallstones which are formed secondary to bacterial and parasitic infections such as *Clonorchis sinensis* or due to stasis of bile. Finally, mixed gallstones account for 80% of gallstones and have a cholesterol content of 20–50% [6, 7].

## Imaging in gallstone-related disease

Multiple imaging modalities play a role in the diagnosis of the broad spectrum of gallstone-related disease including plain radiography, ultrasonography, computed tomography (CT), scintigraphy and magnetic resonance imaging (MRI).

**Fig. 1:**
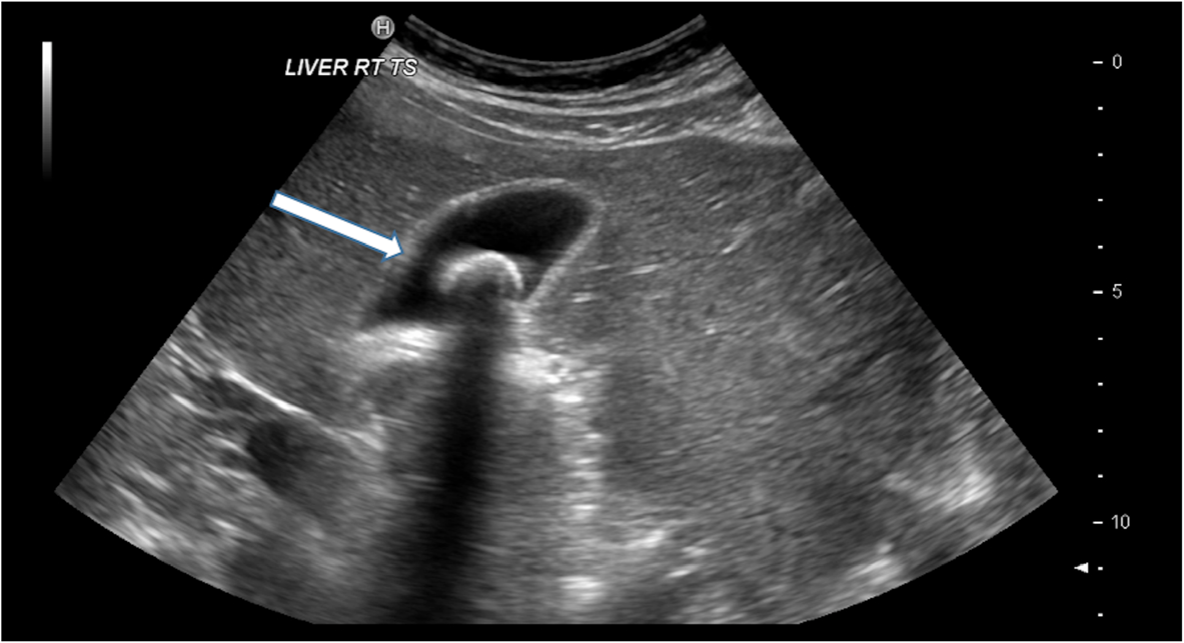
Sagittal ultrasound image of the gallbladder which demonstrates the typical appearance of a gallstone that is hyperechoic with posterior acoustic shadowing (arrow)

Ultrasound is by far the most common and useful imaging modality in assessing gallstones within the gallbladder (cholelithiasis) and associated gallbladder pathology and has the added benefit of no radiation dose to the patient. With ultrasound, gallstones are characteristically echogenic and demonstrate posterior acoustic shadowing regardless of the gallstone composition (Fig. 1). As ultrasound is a bedside dynamic study, stone mobility can be demonstrated with patient manoeuvering, and this can help in differentiating gallstones from gallbladder polyps, which can mimic cholelithiasis sonographically.

Another principle advantage of ultrasound over other imaging techniques in the investigation of acute cholecystitis is the ability to evaluate for a sonographic Murphy’s sign, which can be a reliable indicator of acute cholecystitis with a high sensitivity [8]. Sonographic Murphy’s sign is where the patient reports maximal pain as the sonographer presses over the fundus of the distended gallbladder with the ultrasound probe (and differs to clinical Murphy’s sign).

The major limitation of ultrasound in the imaging of gallstone-related disease is the frequent inability to assess the distal common bile duct (CBD) due to overlying bowel gas. Occasionally, a stone can be seen within the biliary system; however, often the presence of a CBD stone can be inferred from visualised proximal biliary dilatation and clinical presentation with painful jaundice or an obstructive pattern on liver function tests. This requires further work up with alternative imaging.

**Fig. 2:**
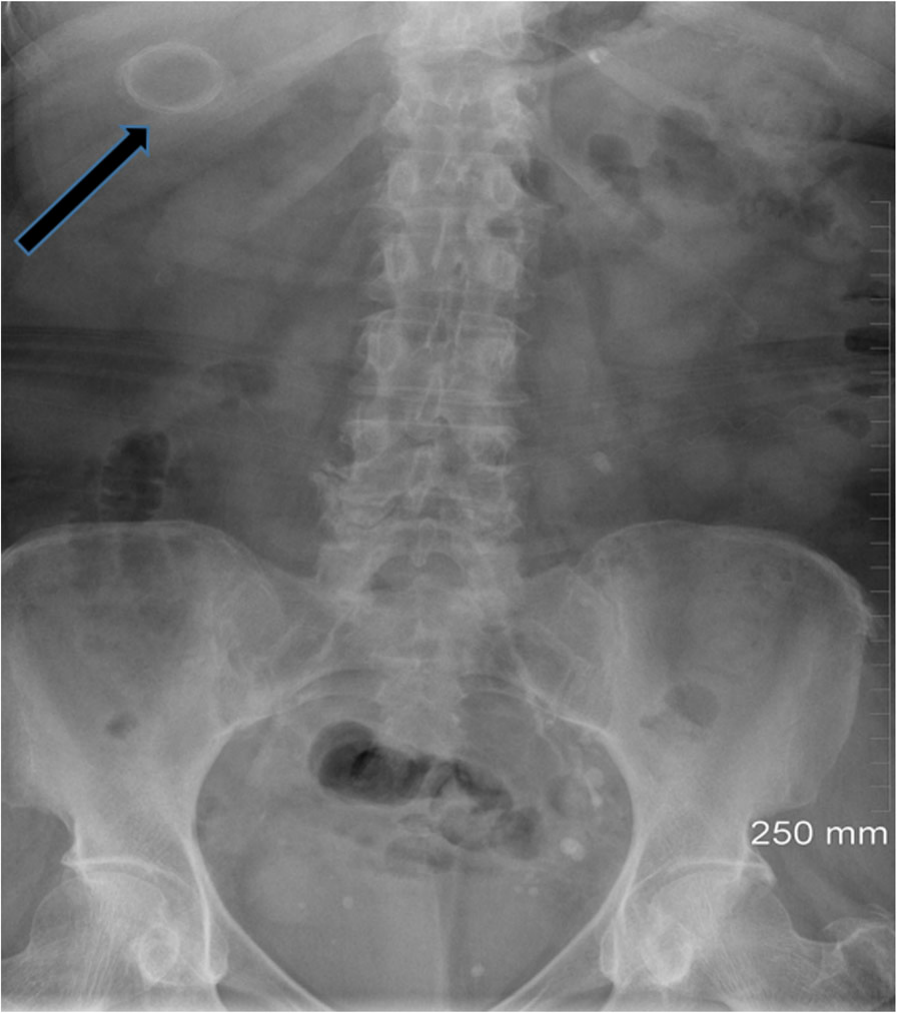
Abdominal radiograph revealing a large rounded calcified density with a central lucency in the right upper quadrant consistent with a peripherally calcified gallstone

Plain radiography is limited in the diagnosis of gallstones as only 15–20% of gallstones are radio-opaque on X-ray [6]. The classical radiographic appearance is the “Mercedes Benz sign” which is an outer radio-opaque rim with a radiolucent centre which is caused by calcification of the gallstone rim and gas fissuring within the gallstone. Calcification of gallstones occurs with increased calcium in bile [9] (Fig. 2).

**Fig. 3:**
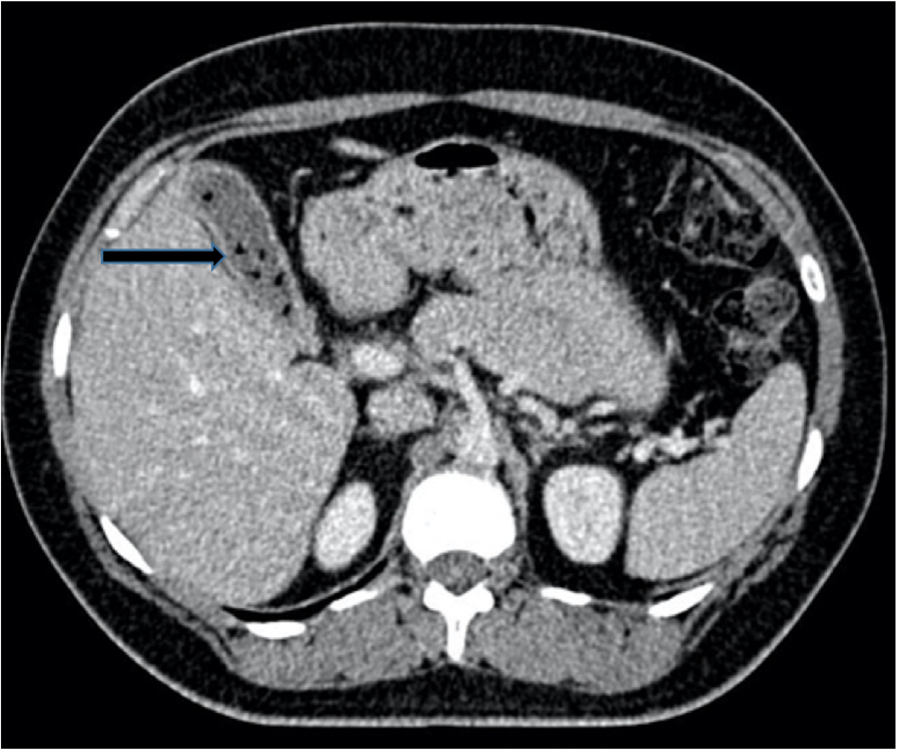
Axial contrast-enhanced CT of the abdomen. There are multiple small gallstones within the gallbladder which are hypoattenuating relative to the surrounding bile

**Fig. 4:**
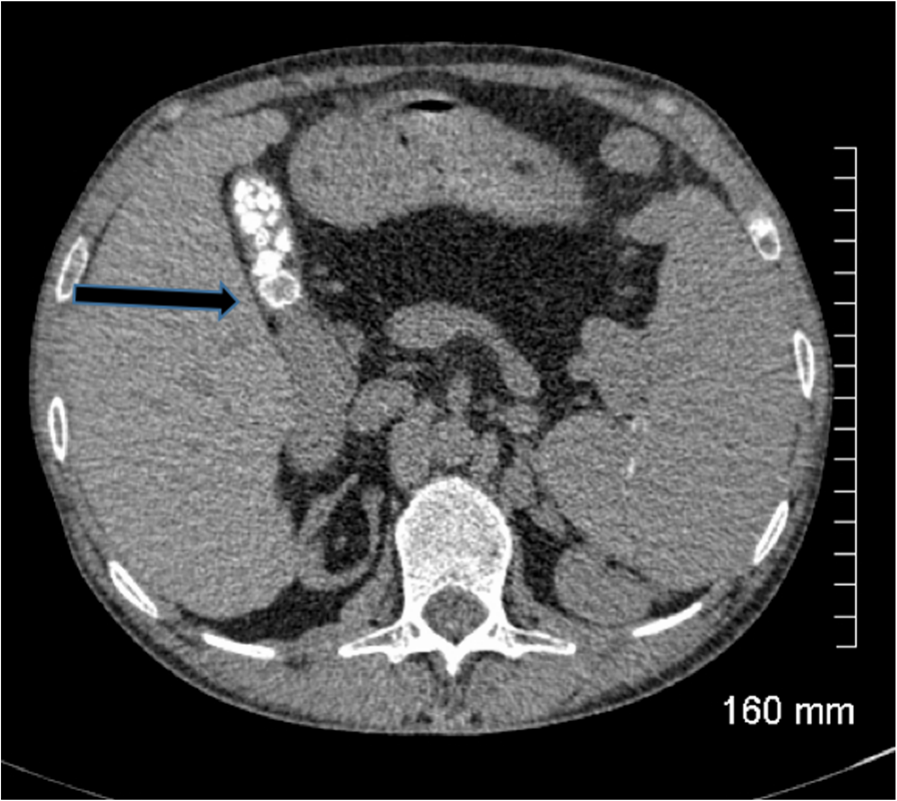
Axial contrast-enhanced CT of the abdomen. There are several calcified gallstones (arrow) within the gallbladder which are hyper attenuating relative to surrounding bile

On CT, a high percentage of cholesterol stones are hypoattenuating relative to bile (Fig. 3) and calcified stones are hyperattenuating relative to bile (Fig. 4); however, a significant proportion of stones are iso-attenuating relative to surrounding bile and may be radiologically occult on CT. Dual-energy CT has been shown to improve detection of gallstones with low KV imaging and base substance imaging, such as calcium-based and lipid-based imaging, which is more sensitive at detecting gallstones than traditional higher KV imaging. Despite these imaging advances, CT remains inferior to ultrasound at assessing the gallbladder and results in a significant radiation dose to the patient. As such, ultrasound is the imaging modality of choice for initial assessment of suspected gallbladder pathology. CT however can be very effective at assessing extra-biliary gallstone pathology and complications arising from gallstone pancreatitis and cholecystitis [8, 10].

**Fig. 5:**
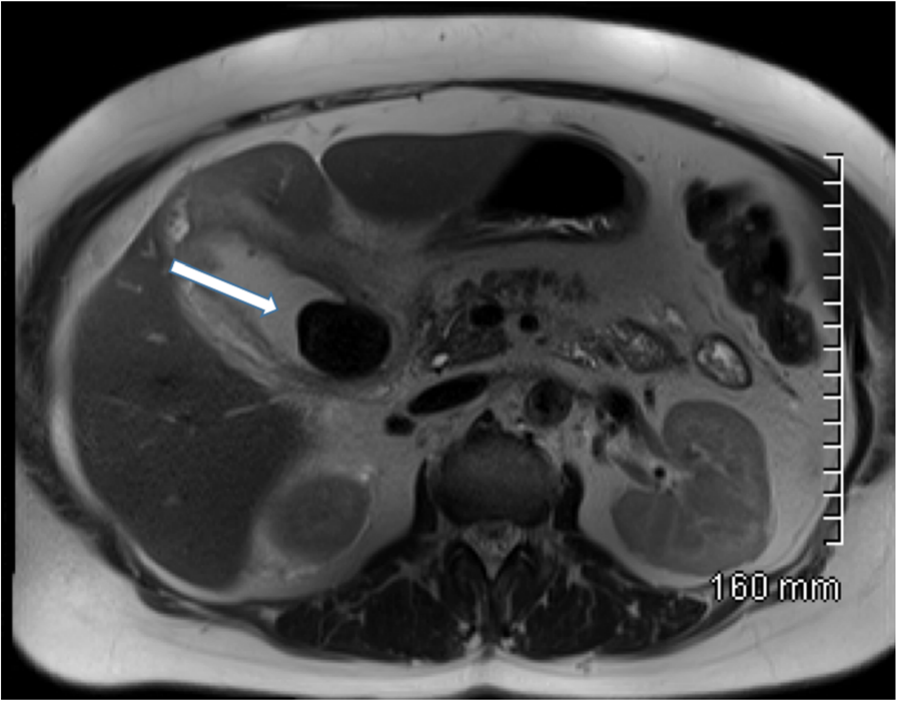
Axial T2-weighted MRI of the abdomen. There is a large gallstone within the gallbladder which is markedly hypointense relative to the surrounding hyperintense bile. The gallbladder wall is thickened with surrounding pericholecystic fluid consistent with acute cholecystitis

MRI is another very effective tool for diagnosing gallstones and associated pathological processes. Magnetic resonance cholangiopancreatography (MRCP) is typically performed using heavily T2-weighted sequences, supplemented by fat-saturated T1- and T2-weighted MRI, and with steady-state gradient-echo acquisitions. Heavily T2-weighted image sequences are particularly helpful in delineating ductal anatomy. Gallstones typically appear as low signal or signal void on T2-weighted imaging surrounded by T2 hyperintense bile [11] (Fig. 5). MRCP has largely replaced endoscopic retrograde cholangiopancreatography (ERCP) as the gold standard for diagnosing choledocholithiasis due to its high sensitivity of 90–94% and specificity of 95–99% without the use of ionising radiation or ERCP-related complications such as pancreatitis which can result in significant morbidity and even mortality [12, 13].

**Fig. 6:**
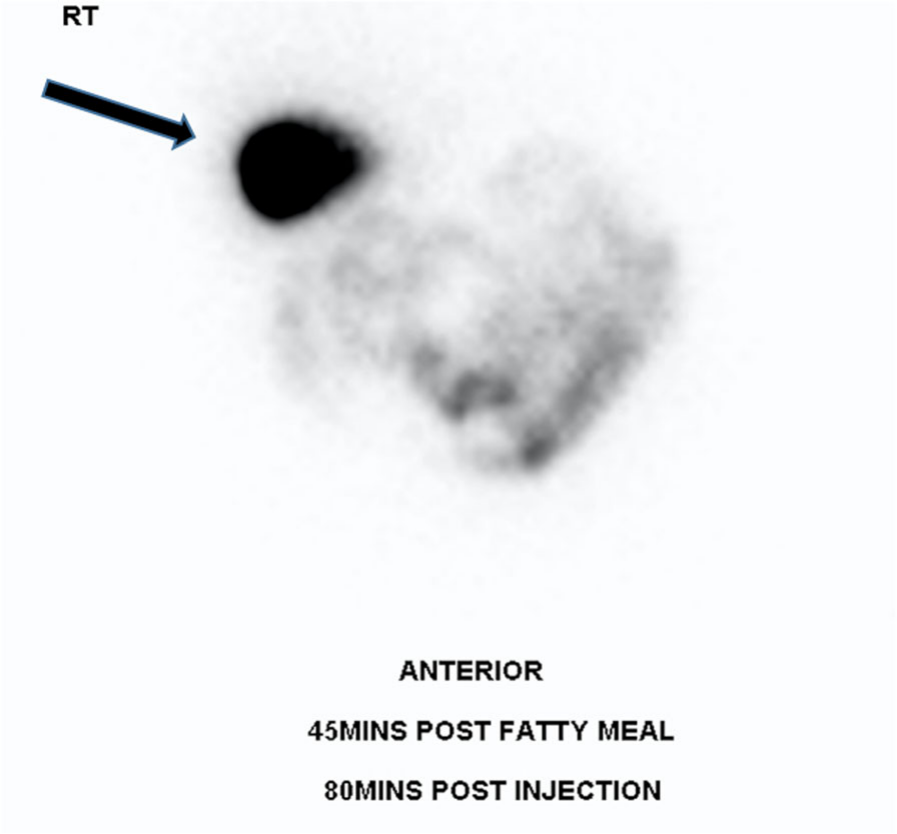
Normal cholescintogram 80 min post-injection of mebrofenin labeled with technetium-99. The radio-isotope is taken up by the liver and excreted into bile. Bile will pool in a non-obstructed normal gallbladder (arrow). In a gallbladder obstructed due to gallstones, the radioactive bile is unable to pool

Nuclear medicine imaging with scintigraphy and with SPECT/CT can be used to dynamically assess the gallbladder. Technicium-99 labeled mebrofenin is administered and taken up by bile producing cells and subsequently excreted into the biliary system. The patient is typically imaged at 1 h and at 4 h post-administration of radioisotope. A normal gallbladder will be well delineated as it fills with radioactive bile. In cases of cholecystitis or gallbladder obstruction due to an impacted stone in the cystic duct, the gallbladder will not be visualised as the radioactive will not accumulate within the gallbladder. If the gallbladder is not visualised, morphine analogues can be given to induce sphincter of Oddi contraction and aid gallbladder filling. Cholescintigraphy for acute cholecystitis has a sensitivity of 97% and a specificity of 94% [14]. This is actually superior to ultrasound; however, this technique is more expensive and time consuming and also confers a radiation dose to the patient and staff and thus is generally not a first-line investigation (Fig. 6).

**Fig. 7:**
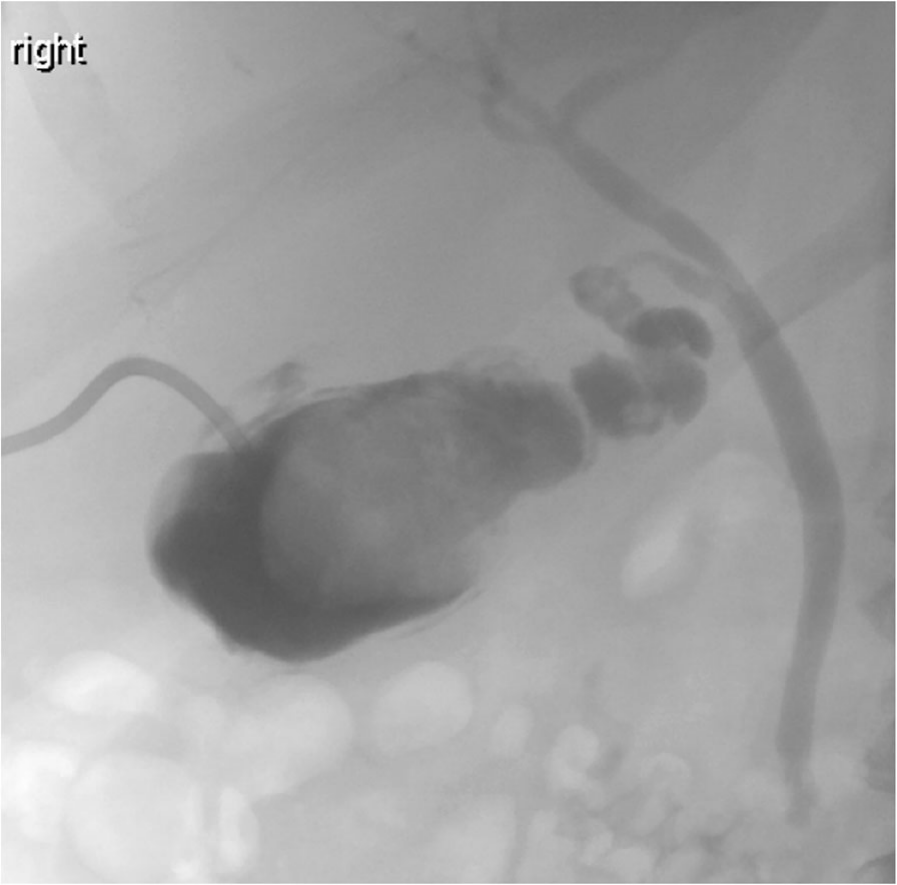
Cholangiogram via a cholecystostomy tube demonstrating a distended gallbladder with a large filling defect consistent with a gallstone. The cystic duct and common bile duct are patent. Contrast is seen in the duodenum

There is an increasing role for interventional radiology (IR) in gallstone-related diseases. Percutaneous cholecystostomy can be used as a temporizing measure in critically ill patients who are too sick to proceed to immediate surgery and cholecystectomy. It is also occasionally being used as a therapeutic measure in the setting of an ageing population with multiple co-morbidities, many of whom may have contraindications to general anaesthesia and surgical intervention. In patients who have had a cholecystostomy placed, cholangiograms can be performed to demonstrate resolution of or ongoing obstruction of the biliary tree by assessing for filling defects (gallstones) within the biliary system (Fig. 7). Percutaneous transhepatic cholangiography (PTC) with biliary stenting is a very effective treatment to decompress the biliary system in the case of obstructing choledocholithiasis, most often in cases not amenable to ERCP. IR also play an active role in the management of the complications of gallstone disease through drain insertion for hepatic or peripancreatic abscesses, in the case of pancreatitis, and for post-operative intra-abdominal collections post-cholecystectomy.

## Gallstone pathology

**Fig. 8:**
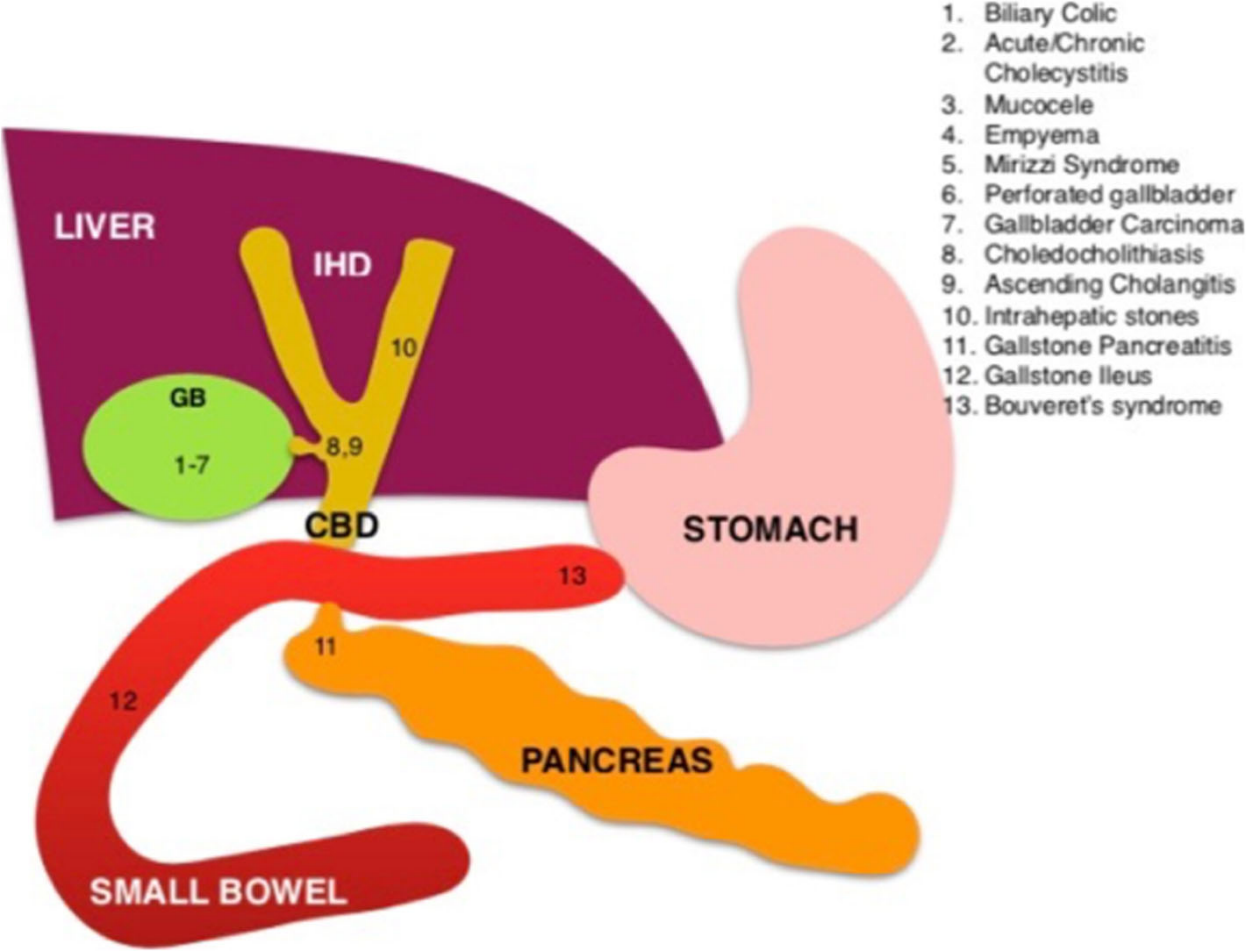
Illustration outlines the multitude of locations within the digestive tract where gallstones can manifest and lists the pathological processes that occur in these locations

The broad spectrum of gallstone-related disease can be broken down based on the anatomical locations in which they occur (Fig. 8).

### Gallbladder

Unsurprisingly, the most common location for gallstones and thus gallstone-related disease is the gallbladder.

Biliary colic is caused when a stone temporarily obstructs the drainage from the cystic duct, resulting in severe cramping abdominal and right upper quadrant pain that can radiate to the back and right shoulder tip as the gallbladder contracts (typically, these symptoms are temporary and subside with resolution of the cystic duct obstruction). Ultrasonography will often demonstrate cholelithiasis without associated complications in patients with simple biliary colic.

**Fig. 9:**
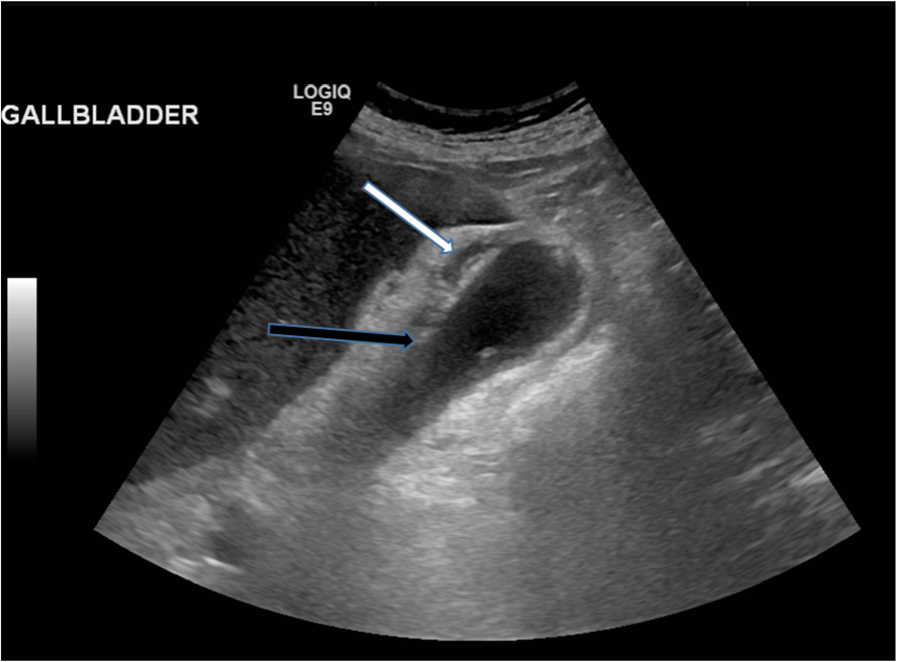
Sagittal ultrasound image of the gallbladder which contains hyperechoic gallstones. There is gallbladder wall thickening (black arrow) and pericholecystic fluid (white arrow) consistent with acute calculous cholecystitis

**Fig. 10:**
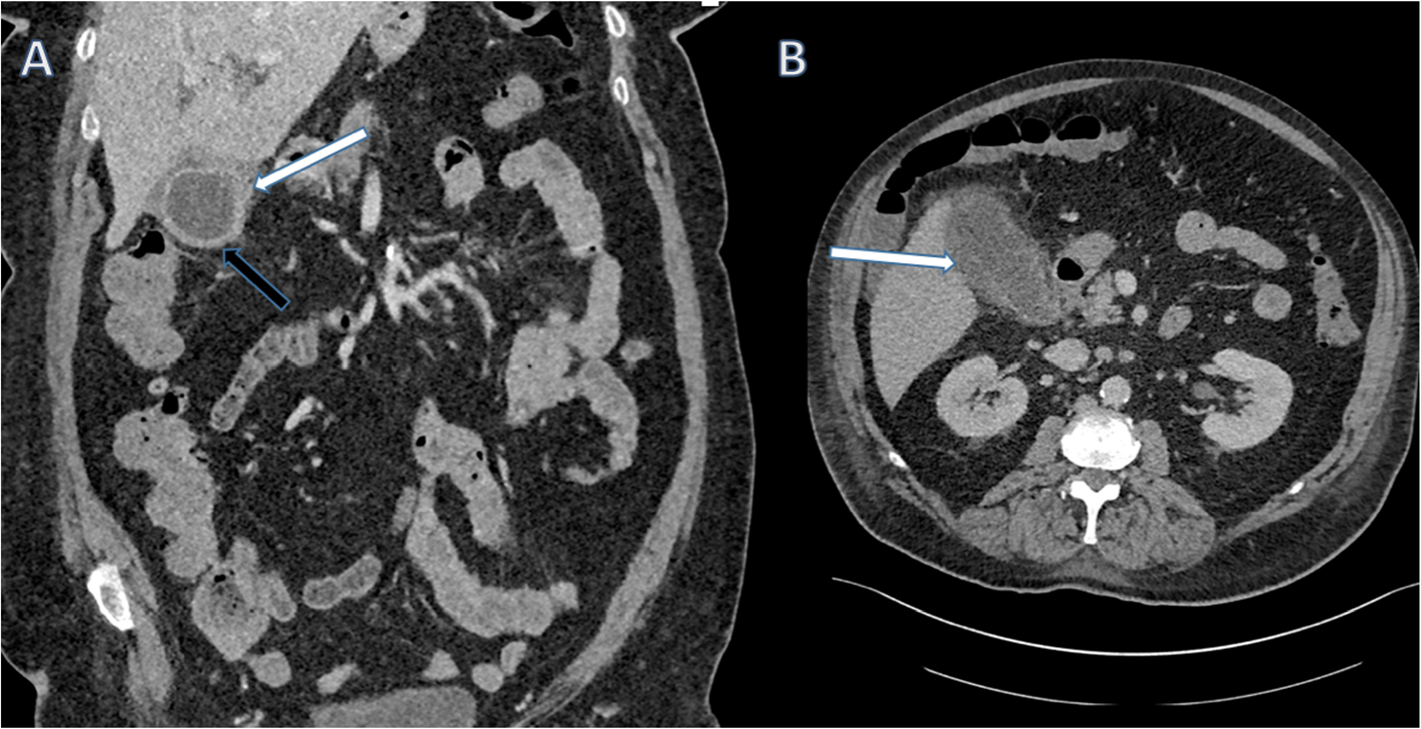
Coronal (**a**) and axial (**b**) contrast-enhanced CT of the abdomen. There is gallbladder wall thickening (black arrow) and pericholecystic fluid (white arrows) consistent with acute cholecystitis

Calculous cholecystitis refers to infection and inflammation of the gallbladder wall caused by irritation from gallstones, and this can be an acute or chronic process. The typical clinical presentation of acute cholecystitis is of right upper quadrant pain, with or without radiation to the right shoulder, which is more constant compared to the intermittent pain seen in biliary colic. There is usually associated pyrexia and other infective symptoms such as nausea and vomiting. The typical imaging features of acute cholecystitis are as follows: gallbladder wall thickening, pericholecystic fluid and a distended gallbladder [8] (Figs. 9 and 10). Chronic cholecystitis is caused by repeated episodes of biliary colic and acute cholecystitis over time, and in contrast to acute cholecystitis, the gallbladder is usually shrunken down and the wall is thickened and scarred.

**Fig. 11:**
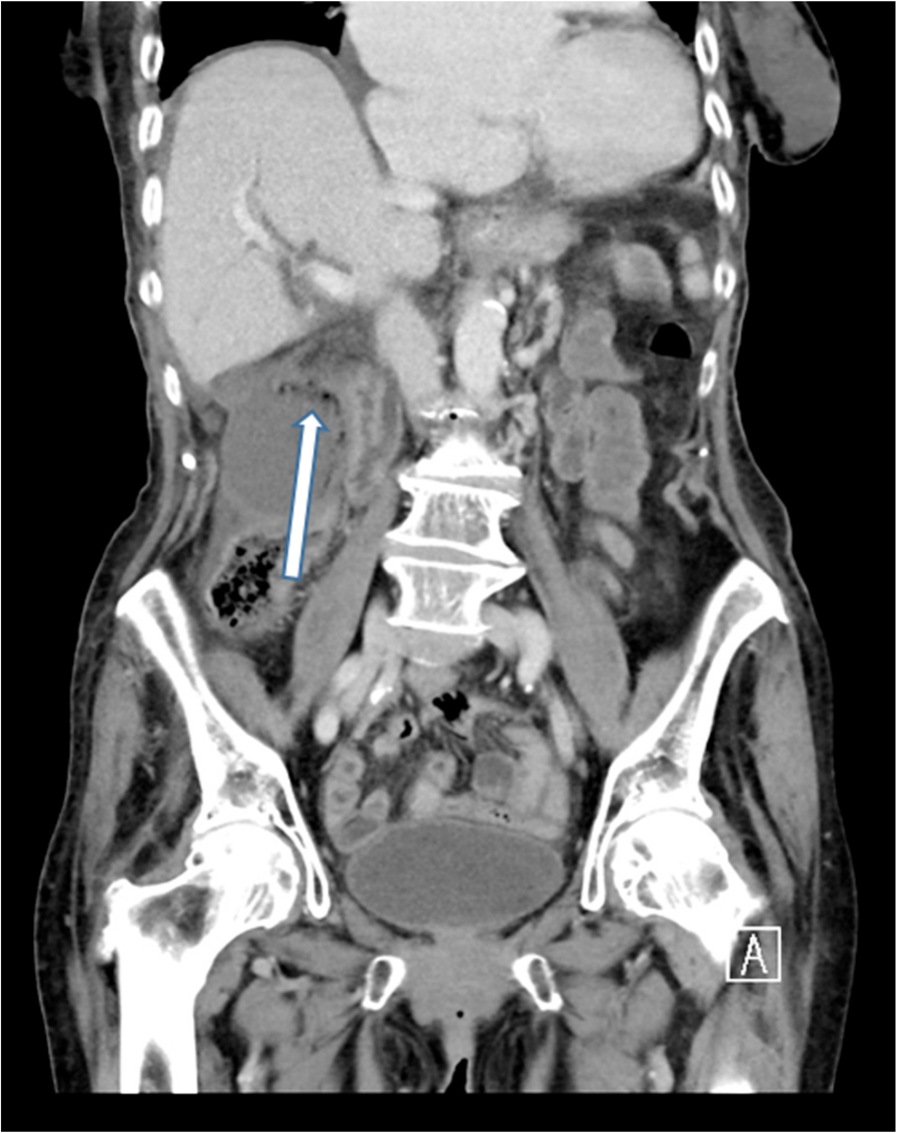
Coronal contrast-enhanced CT of the abdomen demonstrating a markedly distended gallbladder. There are multiple locules of gas within the anti-dependent gallbladder wall (arrow). Findings are consistent with emphysematous cholecystitis

Emphysematous cholecystitis (EC) is a particular entity in which the gallbladder wall becomes necrotic, and this typically occurs with bacterial organisms such as the gas forming organisms such as clostridium, or with E. coli infections. Although rare, EC is associated with high mortality secondary to gallbladder perforation and gangrene, and EC is seen more commonly in patients with diabetes mellitus, coronary artery disease and SIRS (systemic inflammatory response syndrome) [8]. On plain films and CT, gas can be seen within the gallbladder wall (Fig. 11).

**Fig. 12:**
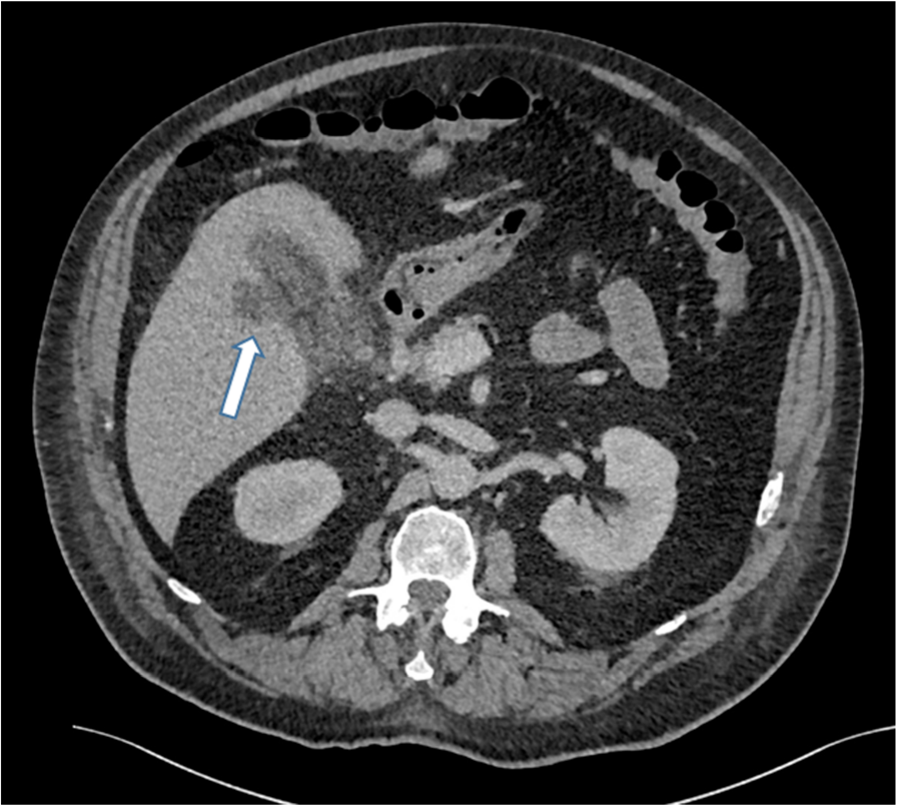
Axial contrast-enhanced CT of the abdomen. There is gallbladder wall thickening and pericholecystic fluid consistent with acute cholecystitis. There is a defect in the medial gallbladder wall with a hypoattenuating collection within segment 5 of the liver (arrow). Appearances are consistent with gallbladder perforation and hepatic abscess

**Fig. 13:**
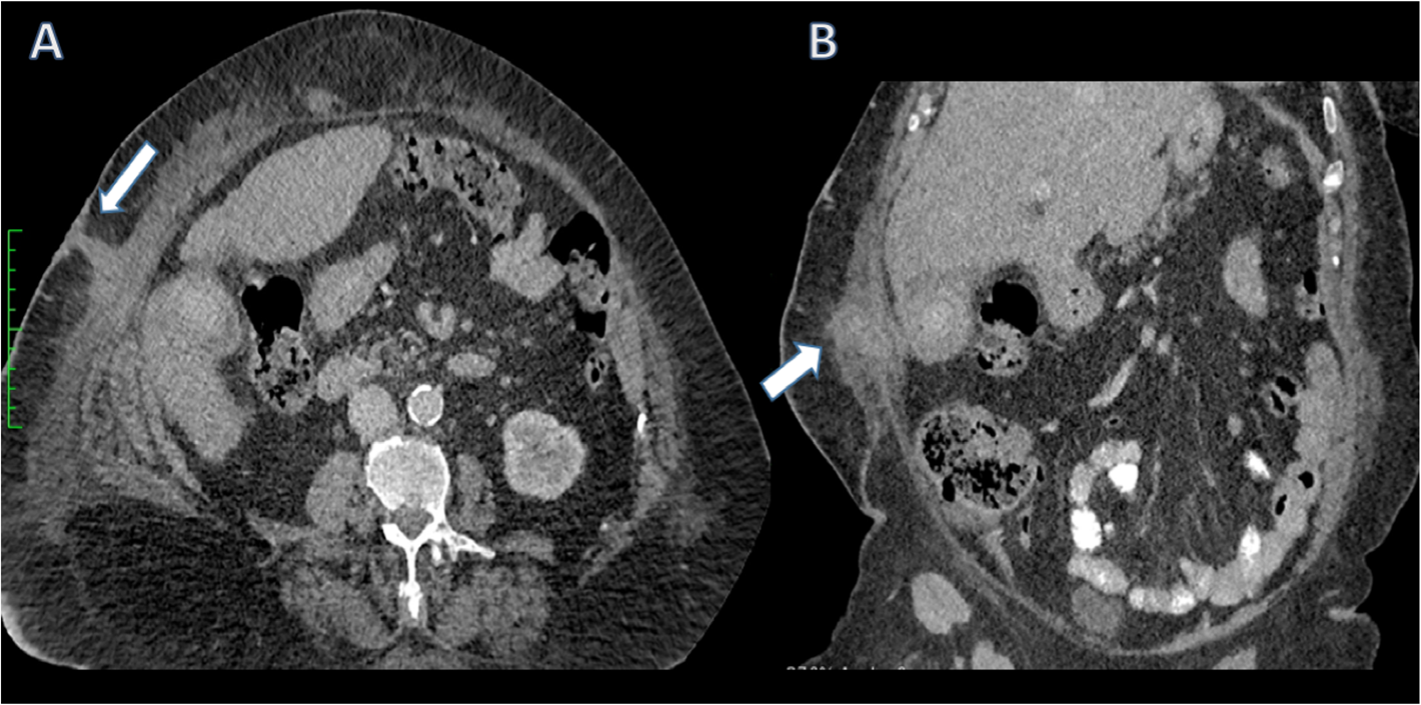
Axial (**a**) and coronal (**b**) contrast-enhanced CT of the abdomen. There is extensive gallbladder wall thickening and stranding with the inflammatory process extending through the peritoneum to the right anterolateral abdominal wall with fistulation to the skin (arrows)

**Fig. 14:**
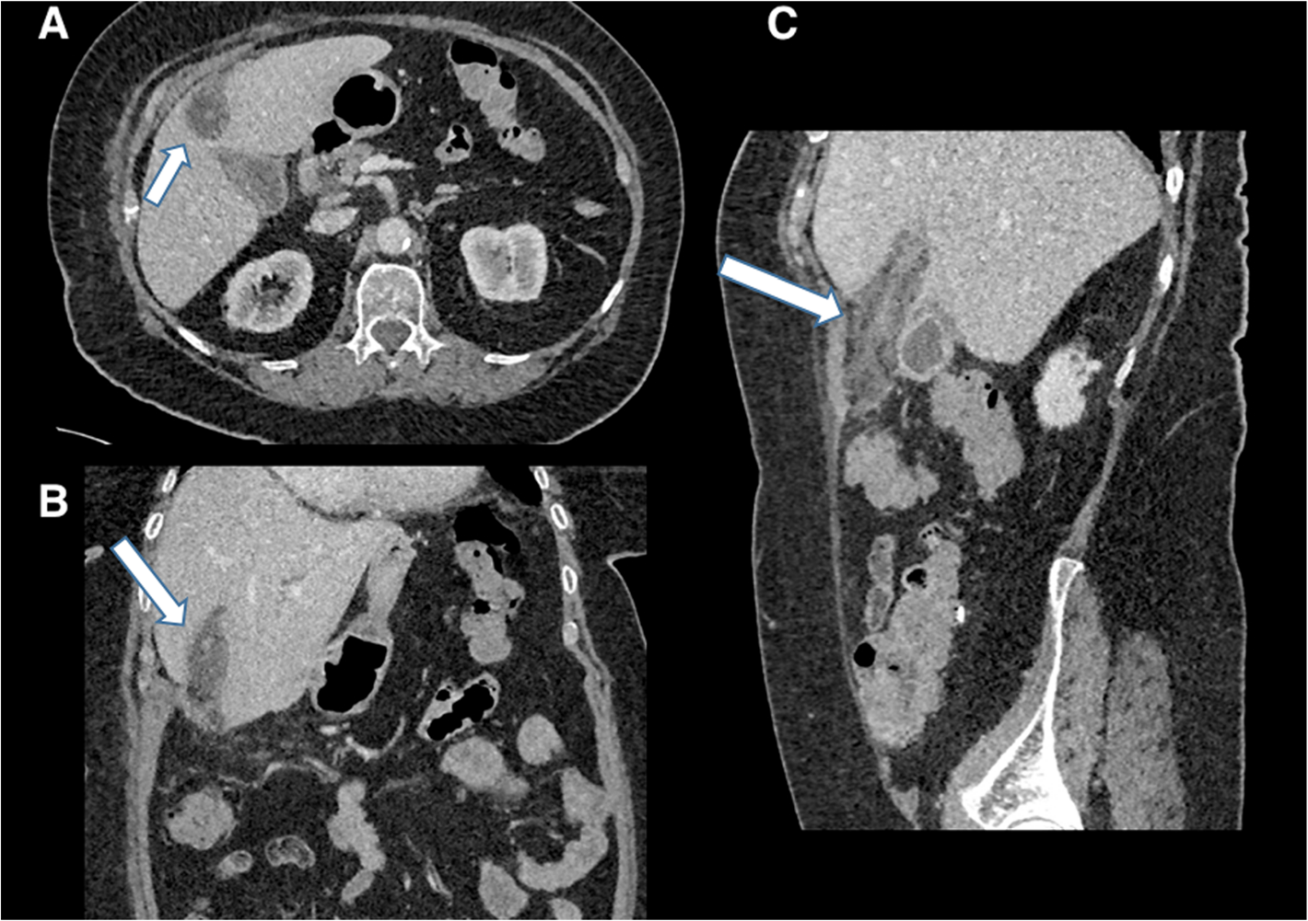
Axial (**a**), coronal (**b**) and sagittal (**c**) contrast-enhanced CT of the abdomen. There is gallbladder wall thickening and pericholecystic fluid consistent with acute cholecystitis. There is marked expansion and oedema surrounding the falciform ligament (arrows). Appearances are due to thrombophlebitis of a recanalised umbilical vein

If an episode of acute cholecystitis is particularly severe or left untreated, it can progress to a gallbladder perforation. This can be appreciated on US or on CT and MRI with pericholecystic abscesses or a defect in the gallbladder wall and a rim of bilious fluid outside of the gallbladder (Fig. 12). There may be an associated intrahepatic abscess which may require radiological or surgical drainage. Other rare complications of severe cholecystitis include cholecysto-cutaneous fistula and thrombophlebitis of a recanalised umbilical vein (Figs. 13 and 14).

**Fig. 15:**
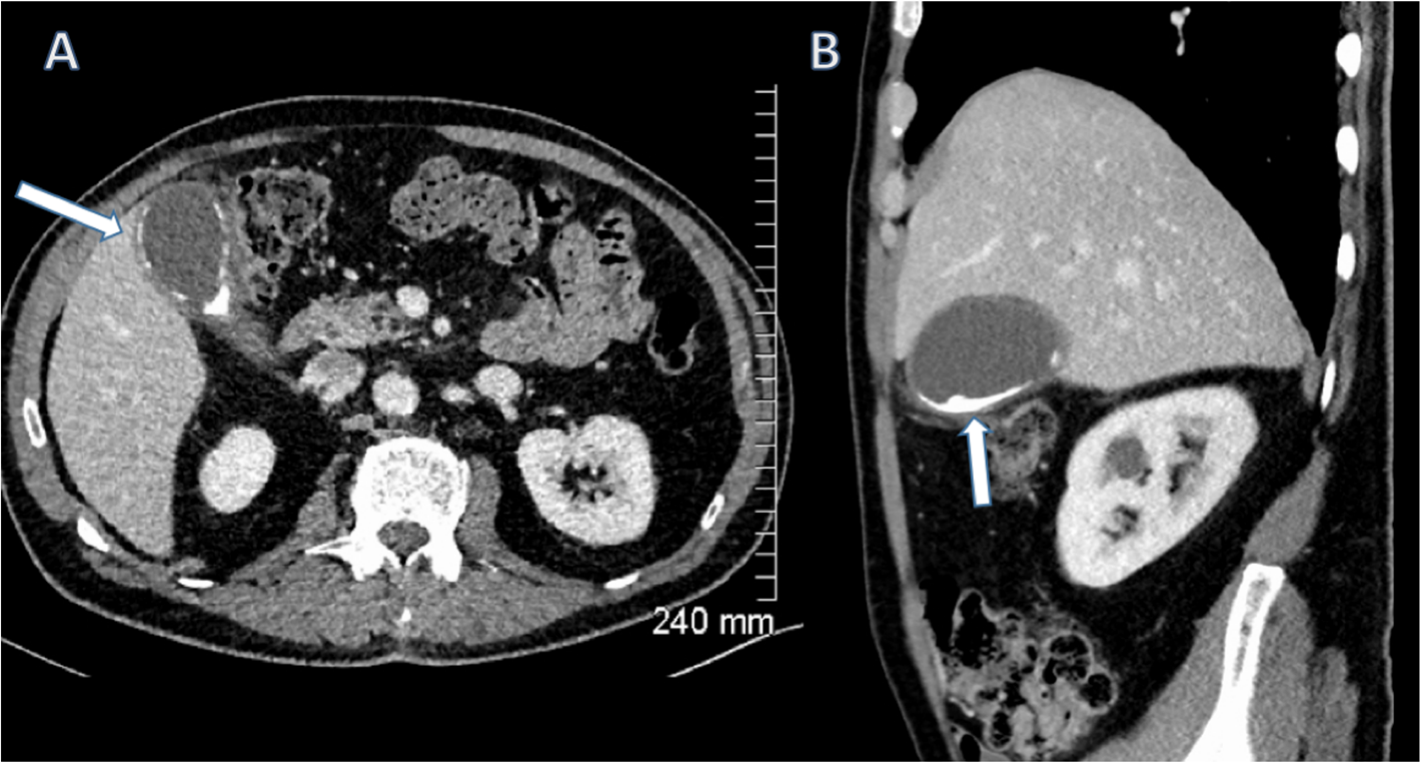
Axial (**a**) and sagittal (**b**) contrast-enhanced CT of the abdomen demonstrating peripheral calcification of the gallbladder wall (arrows) consistent with porcelain gallbladder

**Fig. 16:**
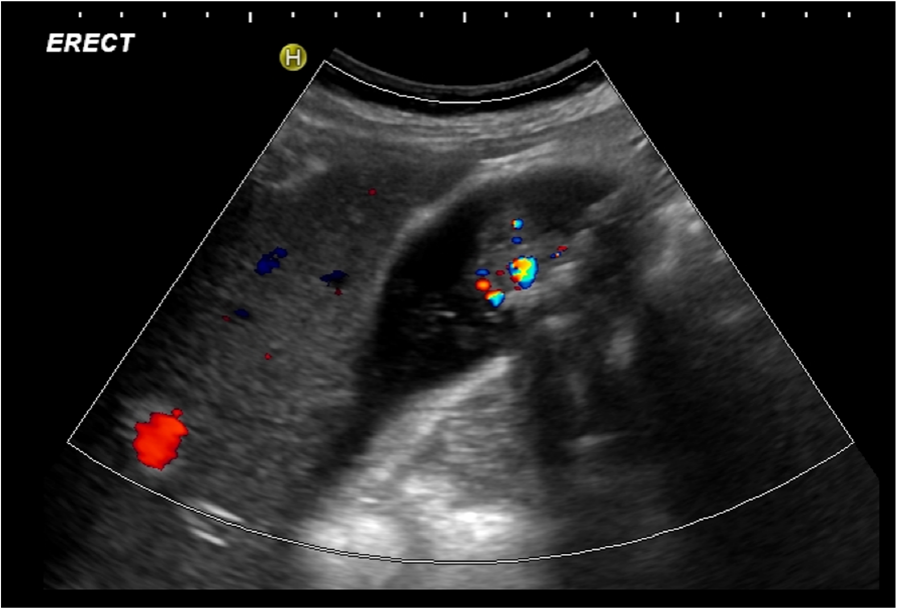
Sagittal ultrasound of the gallbladder demonstrating a soft tissue mass within the gallbladder with internal vascularity consistent with a gallbladder carcinoma

Along the spectrum of chronic cholecystitis is the porcelain gallbladder, where calcification of the gallbladder wall is caused by repeated episodes of cholecystitis (Fig. 15). There is evidence of a causal relationship between gallstones, chronic cholecystitis and gallbladder carcinoma (Fig. 16), and malignancy often presents at an advancedstage; however, definitive proof is lacking [15].

**Fig. 17:**
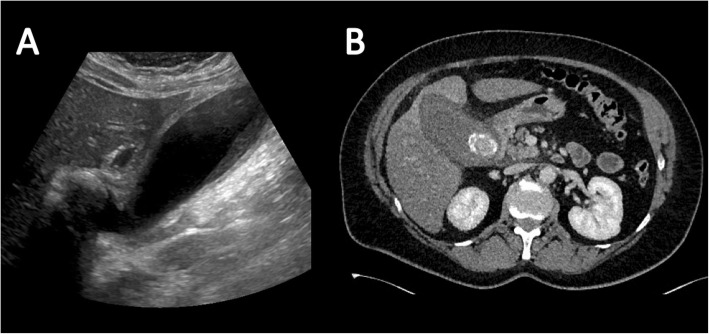
**a** Sagittal ultrasound of a distended gallbladder with an impacted stone in the gallbladder neck. Mild associated gallbladder wall thickening. **b** Axial contrast-enhanced CT of the abdomen in the same patient, again demonstrating a distended gallbladder with an impacted hyperattenuating stone in the gallbladder neck. There is subtle associated fat stranding. Findings are consistent with a gallbladder mucocoele. The gallbladder wall thickening and fat stranding are suggestive of possible empyema

A gallbladder mucocoele results when a stone obstructs the cystic duct causing the gallbladder to become distended with bile. When the bile within the mucocoele becomes infected, this is known as a gallbladder empyema (Fig. 17).

**Fig. 18:**
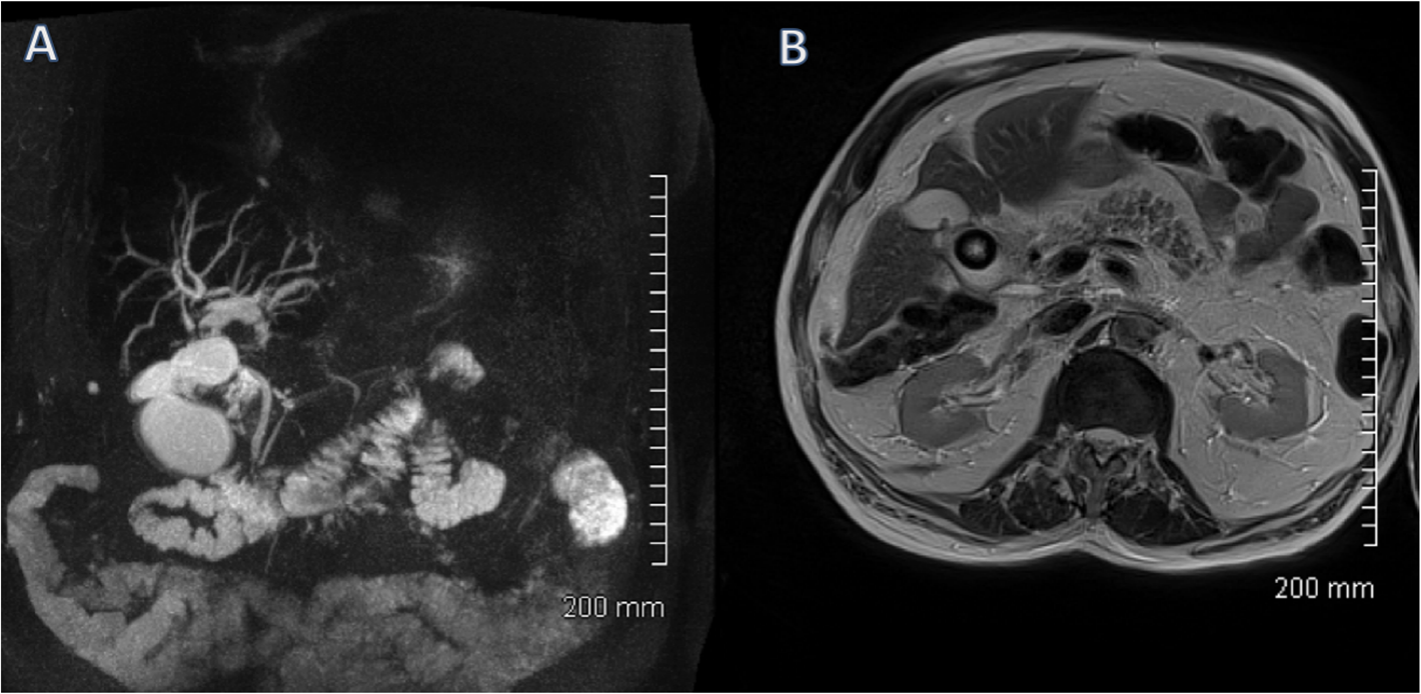
**a** Maximum intensity projection (MIP). **b** Axial T2-weighted MRCP image of the biliary tree. The gallbladder is distended with extensive intrahepatic biliary duct dilatation. The common bile duct is normal in calibre. Appearances are consistent with Mirizzi syndrome, with a stone in Hartmann’s pouch of the gallbladder causing extrinsic compression of the common hepatic duct

Mirizzi syndrome refers to a gallstone that is impacted in the cystic duct or neck of gallbladder which causes extrinsic compression on the common bile duct resulting in obstructive jaundice (Fig. 18).

Both biliary dilatation and the offending gallstone can be seen on ultrasound; however, cross-sectional imaging with MRI or CT, or ERCP, may be needed to confirm that biliary dilatation is secondary to compression from a gallbladder/cystic duct stone rather than secondary to a CBD calculus.

### Pancreaticobiliary system

**Fig. 19:**
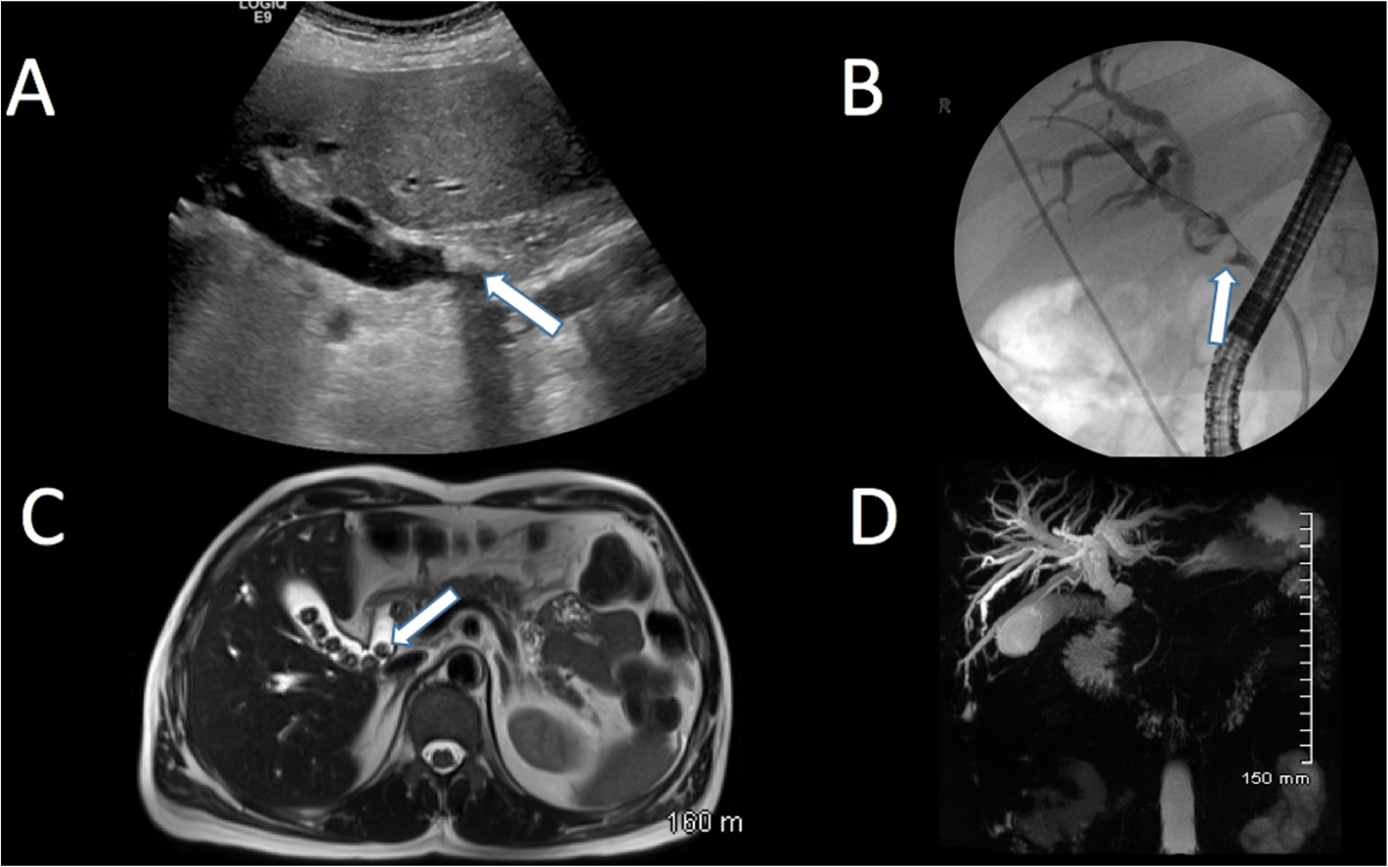
(**a**) Sagittal ultrasound, (**b**) fluoroscopic ERCP cholangiogram, (**c**) T2-weighted axial MRI and (**d**) coronal MIP MRI of the biliary tree. These images demonstrate multiple filling defects (arrows) within the common bile duct with associated biliary duct dilatation consistent with obstructing choledocholithiasis

The next anatomical location where gallstones can be found is outside the gallbladder but within the pancreaticobiliary system. When gallstones exit the gallbladder into the common bile duct (choledocholithiasis), they can often obstruct the normal drainage of bile which can lead to jaundice. This is typically associated with pain, unlike malignant biliary obstruction which is characteristically painless (Fig. 19).

The obstruction of biliary drainage and stasis of bile may result in infection in the form of ascending cholangitis and associated sepsis. The clinical picture associated with this is described in Reynolds’ pentad consisting of fever, right upper quadrant pain, jaundice, hypotension and altered mental status. These patients may require urgent decompression of the biliary system.

In rare cases, there can be retrograde passage of gallstones into the common hepatic duct or the right or left main hepatic ducts, or stones can form in intrahepatic ducts due to biliary stasis.

**Fig. 20:**
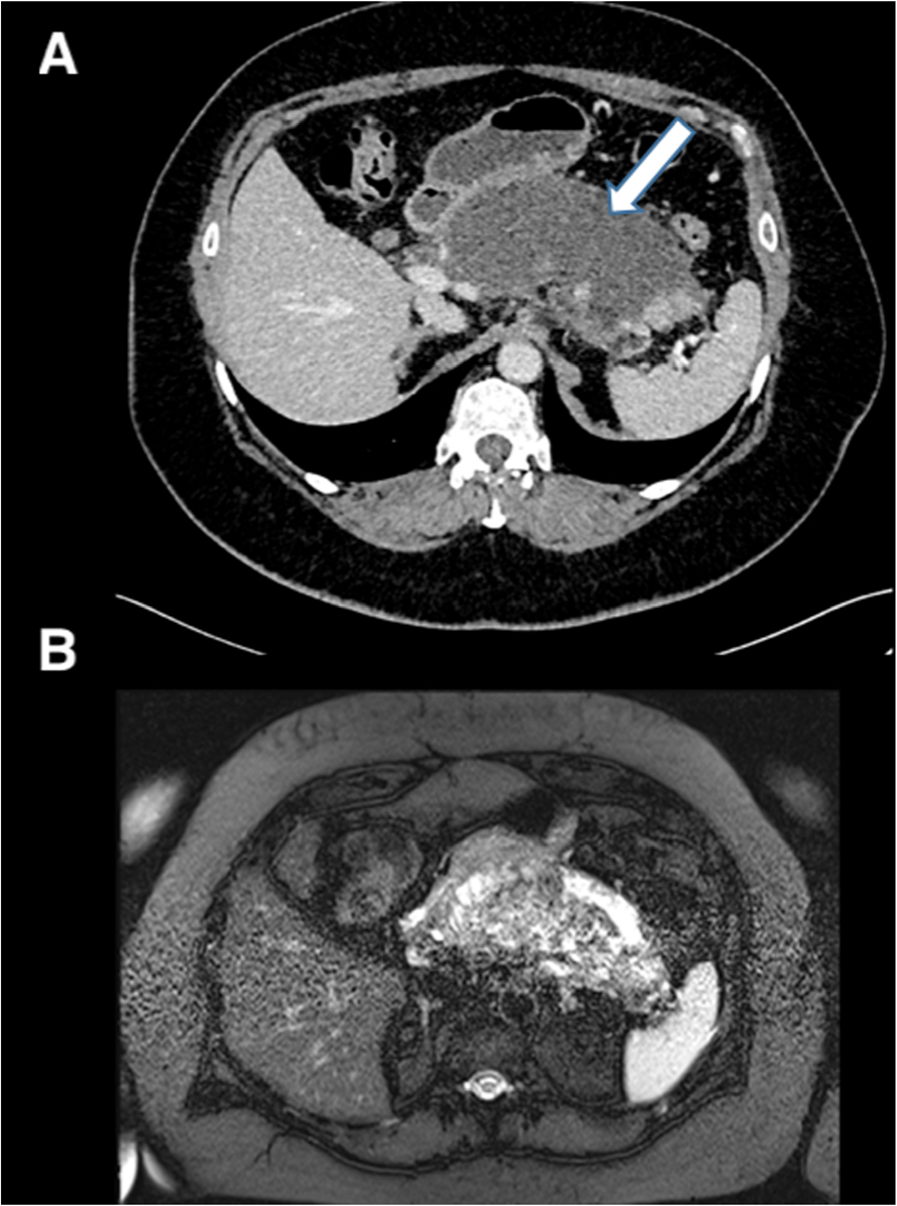
Axial contrast-enhanced CT of the abdomen (**a**) and axial fat-suppressed T2-weighted MRI of the abdomen (**b**) in the same patient demonstrating extensive inflammation and oedema of the pancreas secondary to gallstone pancreatitis with a peripancreatic collection (arrow)

If a gallstone passes down the common bile duct and comes to rest at the ampulla of Vater, it may block the drainage of the pancreatic duct causing back pressure on the pancreatic cells and resulting in gallstone pancreatitis. These patients present with epigastric pain radiating to the back and the severity ranges from mild to severe. There is a significant mortality associated with severe pancreatitis, and critically ill patients should be managed in a high dependency or intensive care monitored environment. While imaging is not usually required or indicated to confirm the diagnosis of acute pancreatitis, an ultrasound of the gallbladder can confirm or rule out the presence of gallstones. CT abdomen/pelvis is best performed 48 h after the onset of symptoms to assess for complications of pancreatitis such as peripancreatic collections or pancreatic necrosis (Fig. 20).

In general, larger gallstones are more likely to obstruct higher in the common bile duct, and as such are more likely to cause obstructive jaundice or cholangitis. Smaller gallstones are more likely to cause pancreatitis as they more freely pass down to the level of the ampulla of Vater [4, 5].

### Extra-biliary complications

Gallstones can also cause pathology outside of the biliary system. The most common cause, although rare, is a cholecystoenteric fistula. Chronic irritation from a large gallstone can erode through the gallbladder wall with fistulisation into small bowel. This can be seen on imaging with air seen within the gallbladder or biliary tree (pneumobilia).

**Fig. 21:**
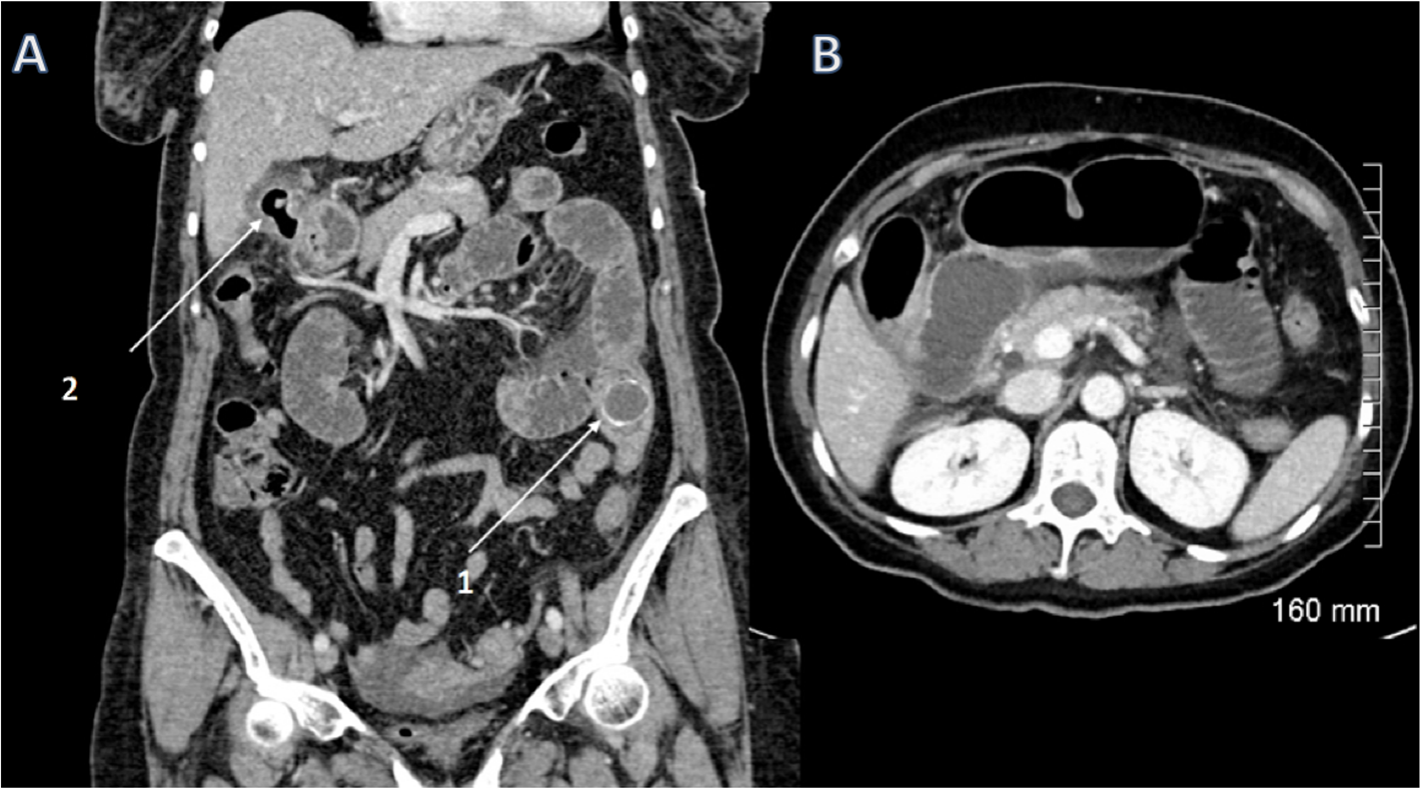
Coronal (**a**) and axial (**b**) contrast-enhanced CT of the abdomen demonstrating multiple dilated loops of small bowel. There is a 3-cm peripherally hyperattenuating obstructing gallstone in the left flank (arrow 1). There is an extensive inflammatory process in the gallbladder bed with air in the gallbladder (arrow 2) consistent with a cholecystoenteric fistula. Appearances are consistent with a bowel obstruction secondary to a gallstone ileus

When a gallstone passes through the fistula into the small bowel, this can result in intestinal obstruction, either proximal or more commonly distal. The most common place for distal small bowel obstruction and gallstone ileum is at the level of ileocecal valve as this is the narrowest point; however, gallstone ileus can occur anywhere in the gastrointestinal tract. The diagnosis is suggested on abdominal X-ray by the presence of pneumobilia in the right upper quadrant with dilated loops of bowel consistent with bowel obstruction. Gallstone ileus is more accurately diagnosed with CT which may show pneumobilia or may directly demonstrate the presence of a cholecystoenteric fistula and associated bowel obstruction (Fig. 21).

**Fig. 22:**
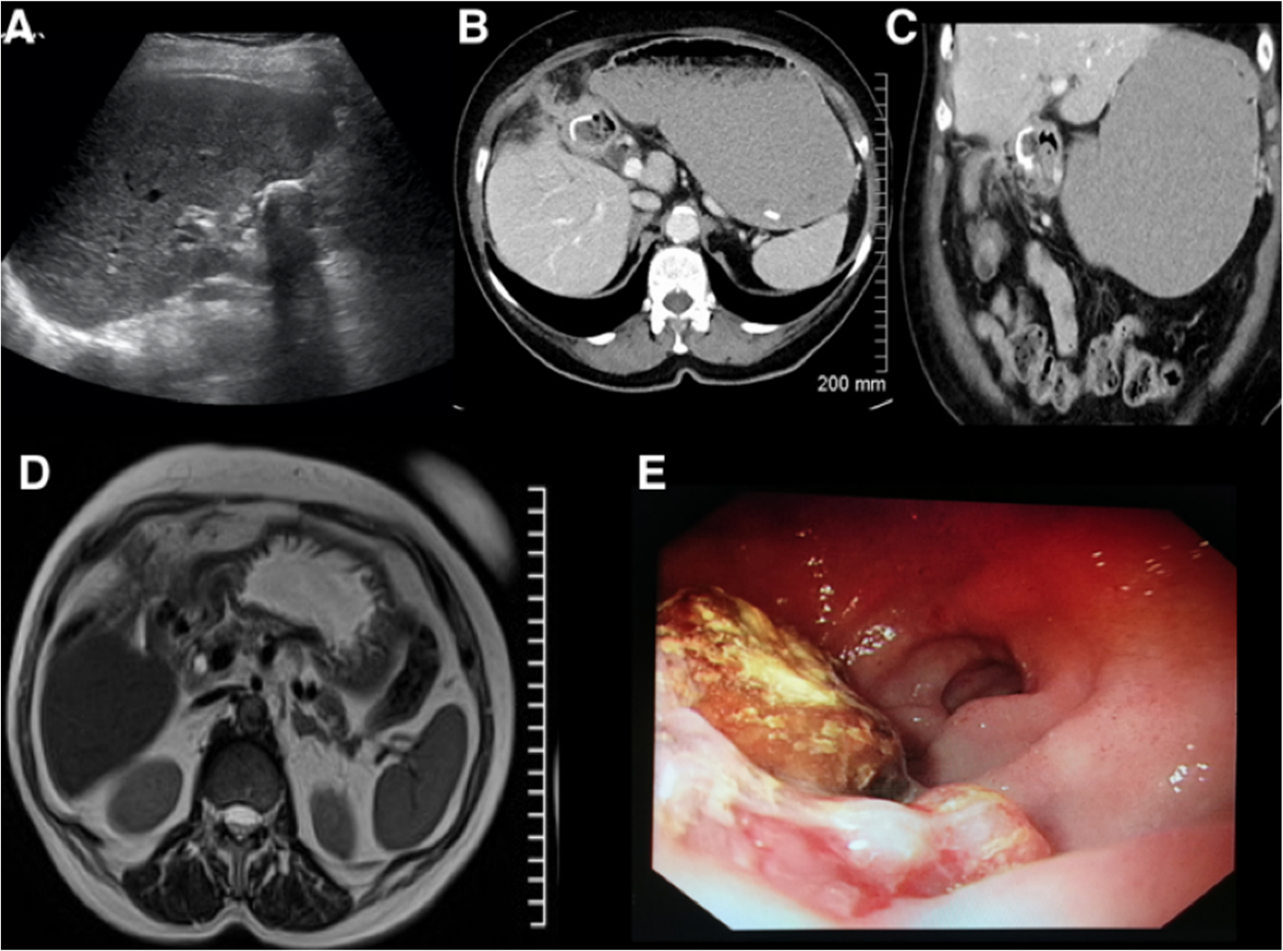
Ultrasound (**a**), CT (**b**, **c**), MRI (**d**) and endoscopic images (**e**). Demonstrating a large calcified gallstone in the proximal duodenum with a massively dilated stomach. Findings are consistent with a proximal bowel obstruction consistent with Bouveret’s syndrome

Bouveret’s syndrome is a particular eponymous syndrome in which a stone obstructs the upper GI tract proximally at the level of duodenum or gastric outlet. Patients typically present with copious vomiting owing to the proximal level of obstruction. There may be little or no small bowel dilatation; in particular, the X-ray abdomen may be completely normal which can falsely reassure. Imaging will demonstrate evidence of gastric outlet or duodenal obstruction related to a gallstone in the upper GI tract (Fig. 22).

### Post-surgery/cholecystectomy complications

**Fig. 23:**
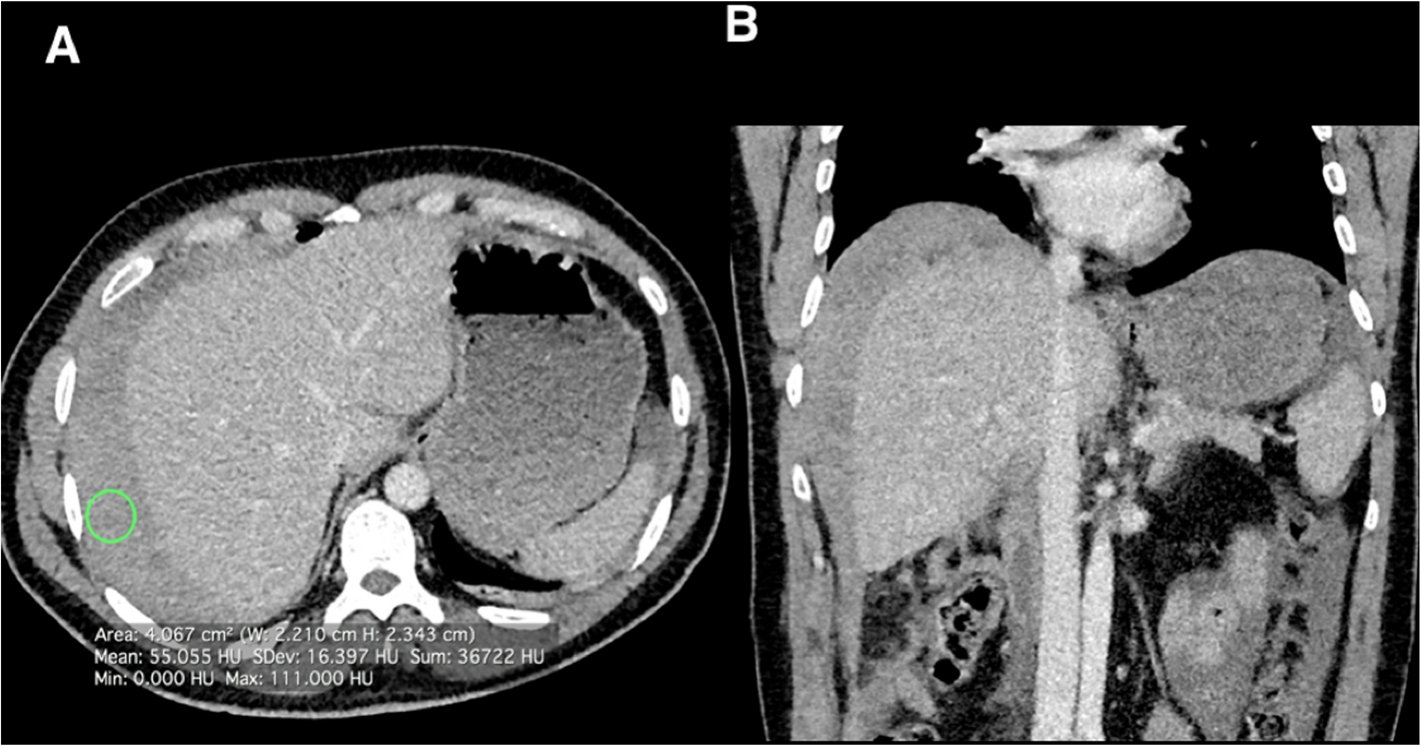
Axial and coronal contrast enhanced CT of the abdomen in a patient several hours post-cholecystectomy. There is large volume perihepatic fluid with an average Hounsfield unit of 55 consistent with post-cholecystectomy bleeding

Finally, there are a number of imaging features post-cholecystectomy that the radiologist should be aware of. Immediate complications can include post-operative bleeding or an injury to the common bile duct resulting in a bile leak and subsequent biloma. CT is the optimal imaging modality for the initial imaging of post-operative complications, where these complications and fluid collections are well appreciated. It can be difficult to differentiate between blood and bile on CT, and measuring a region of interest to obtain the Hounsfield attenuation value of the fluid can help differentiate between the two. The typical Hounsfield unit of blood is 25–75 and that of bile is usually < 20; however, there can be some overlap. Other factors should be considered to ascertain the aetiology of any visualised collection, for example, a layering haemotocritl level with altered attenuation values can be a feature seen with haemorrhagic collections where the inferior denser (haemorrhagic) component is seen dependently [16] (Fig. 23).

**Fig. 24:**
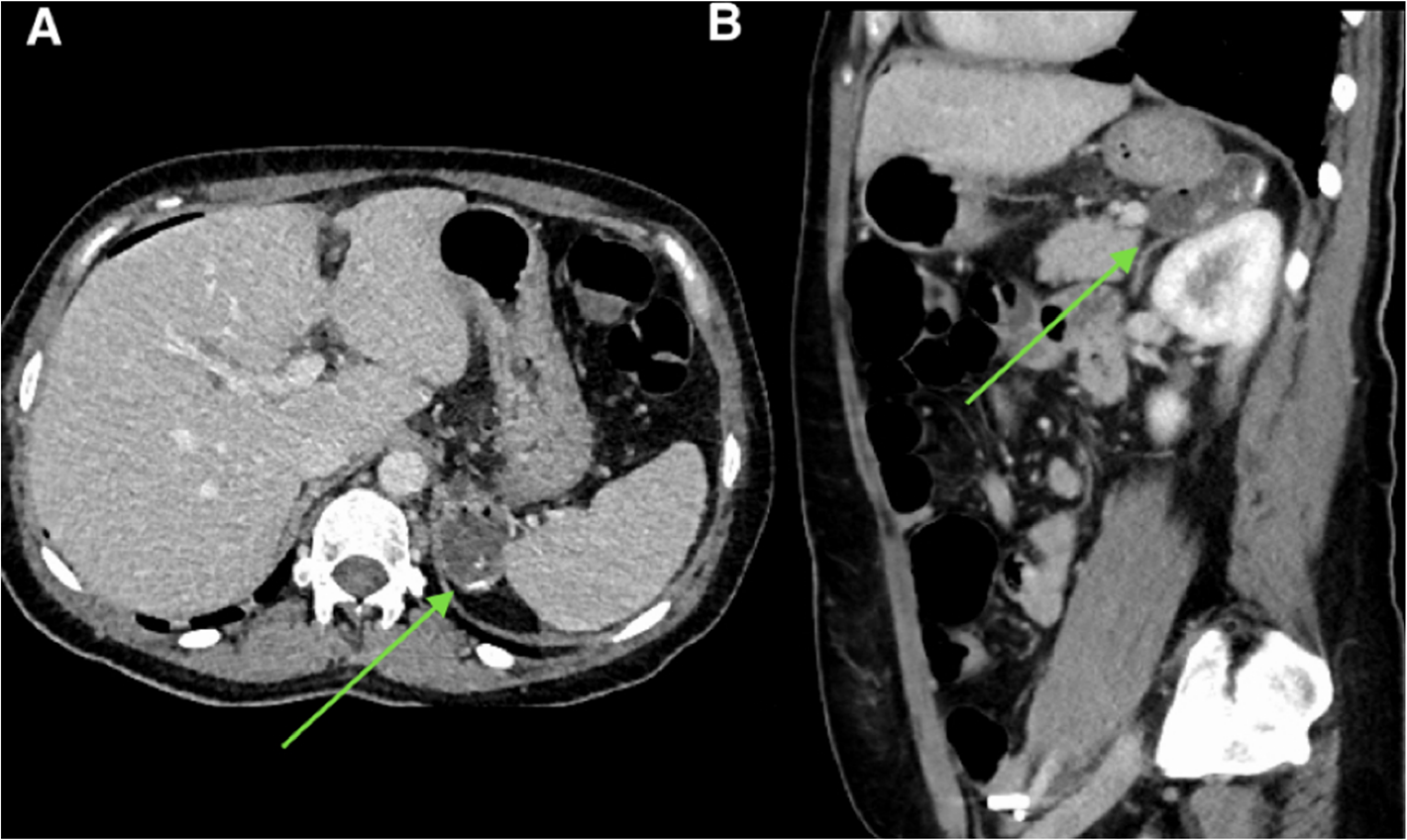
Axial and sagittal images of a contrast-enhanced CT abdomen in a patient several days post-laparoscopic cholecystectomy. There is a rim-enhancing fluid collection compatible with an abscess which contains multiple (dropped) gallstones

Dropped gallstones at time of laparoscopy can have a delayed presentation with post-operative complications such as intrabdominal abscess formation and CT demonstrating a radio-opaque gallstone surrounded by abscess (Fig. 24). Gallstone abscesses without radiopaque gallstones can pose a particular diagnostic challenge as the nidus for infection is not definitely confirmed on imaging. Abscesses related to dropped gallstones can be complex and may extend through abdominal planes and extend extra-peritoneally into adjacent subcutaneous and soft tissue plains. The clinical history will often include a history of prior or difficult cholecystectomy. Gallbladder clips or an absent gallbladder can be seen on cross-sectional imaging as clues.

**Fig. 25:**
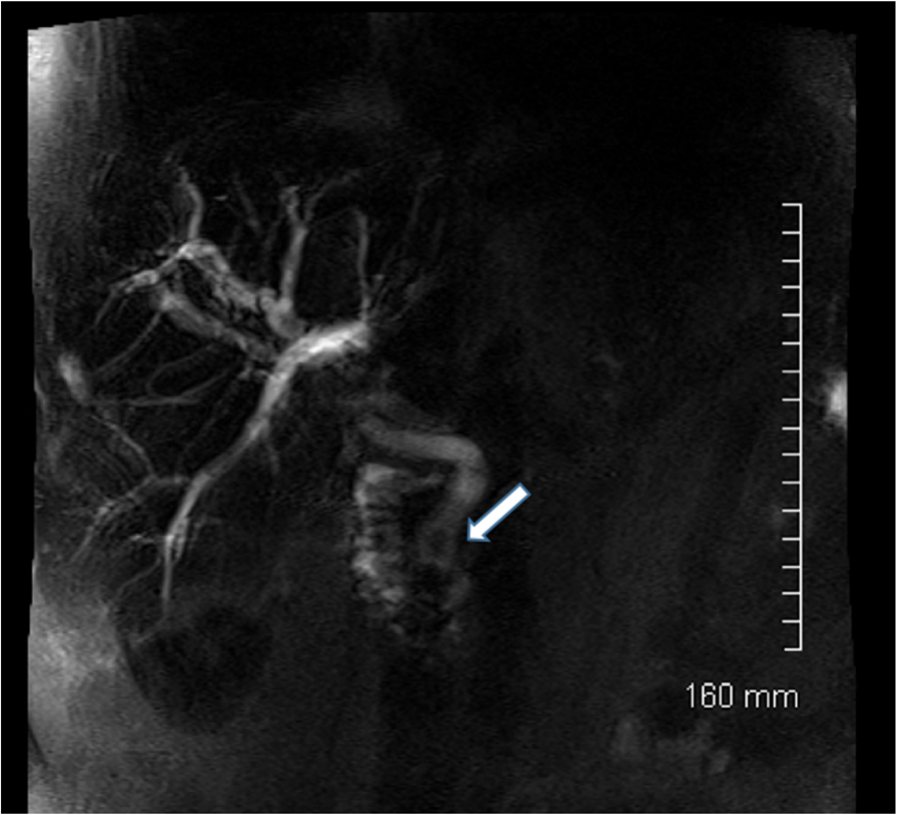
Coronal magnetic resonance MIP image of the biliary tree. The gallbladder is absent consistent with a prior cholecystectomy. There is a filling defect in the distal common bile duct (arrow) with associated biliary duct dilatation consistent with an obstructing gallstone

Patients with occult choledocholithiasis that proceed to cholecystectomy can present with obstructive jaundice and cholangitis in the post-operative period. It is important that any patient in whom choledocholithiasis is suspected undergo MRCP prior to surgery. Alternatively, an intra-operative cholangiogram or choledochoscope can be performed intra-operatively to ensure the common bile duct is clear of stones. Late post-cholecystectomy complications can include stump cholecystitis or a retained cystic duct stump or common bile duct stone. These findings result from incomplete cholecystectomy and can be identified on imaging [17] (Fig. 25).

## Conclusion

Radiologists should be familiar with the wide range of pathological processes that can be seen secondary to gallstones in order to aid prompt diagnosis, treatment and intervention

It is hoped that through understanding the role of multimodality imaging and understanding the anatomic locations of the manifestations of gallstone-related disease, this review paper will assist the radiologist in diagnosing common and less common manifestations of gallstone-related pathology.

## Data Availability

Not applicable.

## Abbreviations

CBD: Common bile duct
CT: Computed tomography
EC: Emphysematous cholecystitis
ERCP: Endoscopic retrograde cholangiopancreatography
IR: Interventional radiology
MRCP: Magnetic resonance cholangiopancreatography
MRI: Magnetic resonance imaging
SIRS: Systemic inflammatory response syndrome
